# Bilateral anterior tibial artery entrapment by the ankle extensor retinaculum: case report

**DOI:** 10.1590/1677-5449.200026

**Published:** 2020-09-21

**Authors:** Ana Luiza Dias Valiente Engelhorn, Anna Luiza Cauduro de Miranda, Luiz Eduardo Biglia, Rafaella Castilho, Sarah Folly Polonio Machado, Maurício Henrique Abrão, Carlos Alberto Engelhorn

**Affiliations:** 1 Pontifícia Universidade Católica do Paraná – PUC-PR, Curitiba, PR, Brasil.; 2 Angiolab, Curitiba, PR, Brasil.; 3 Hospital Marcelino Champagnat – HMC, Curitiba, PR, Brasil.

**Keywords:** anterior tibial artery, intermittent claudication, arterial compression

## Abstract

Vascular entrapment is rare. In the lower limbs it is generally asymptomatic, but may cause atypical intermittent claudication in young people without risk factors for atherosclerosis and inflammatory diseases. The most common type of compression involves the popliteal artery, causing symptoms in the region of the infra-patellar muscles. When discomfort is more distal, other entrapment points should be considered, such as the anterior tibial artery. This article reports the case of a patient with intermittent claudication in both feet due to extrinsic compression of the anterior tibial artery bilaterally by the extensor retinaculum of the ankle, diagnosed by vascular ultrasonography and angiotomography during plantar flexion maneuvers. The patient was treated surgically, resulting in improvement of clinical symptoms.

## INTRODUCTION

Lower limb claudication is classically defined as a difficulty walking and generally affects elderly people who have chronic obstructive vascular disease or degenerative orthopedic diseases. When it affects young people with no obvious vascular or musculotendinous disease detectable in their clinical history or by physical examination, it is considered atypical claudication (AC).^1^ In these cases, arterial compressive phenomena should be suspected.

Vascular entrapment in the lower limbs can be a cause of AC in young people with no risk factors for atherosclerosis. Popliteal artery entrapment (PAA) is the most prevalent of these conditions, with an incidence in the range of 0.16 to 3.5%, has congenital or functional etiology, affects young people, primarily males, causes symptoms involving muscle groups below the knee, and is generally unilateral and asymptomatic.^2^^-^5 However, when the site of effort-related discomfort is more distal, located in the dorsal or plantar part of the foot, we must consider other sites of arterial compression, lower than those typically found in PAA. In these situations, anterior tibial artery entrapment (ATAE) should be suspected. Anterior tibial artery entrapment is a rarely-diagnosed condition.

The objective of this study is to report a case of atypical claudication in a young patient whose vascular arterial physical examination was normal at rest and who was diagnosed by ultrasound with bilateral ATAE caused by the extensor retinaculum of the ankle.

## CASE REPORT

The patient was a 34-year-old male, non-smoker, who worked as a motorcycle delivery rider, and usually practided martial arts fighting. He complained of strong intensity pains in the left ankle and foot, with onset 7 years previously, and with progressive and limiting characteristics. Over time, he began to suffer pain in the right foot as well, but rarely simultaneously. He described the pain as extreme tiredness, similar to cramps, triggered by physical effort, such as martial arts and riding his motorcycle. Conversely, he was able to walk long distances without feeling any pain. To begin with, he was able to achieve relief by ceasing the activity or taking over-the-counter analgesic medications. He had not noticed any other symptoms, such as changes to the temperature or color of the limbs.

Over time, the condition compromised the patient’s quality of life, affecting his personal, professional, and social life. He stopped participating in sports and no longer rode his motorcycle, because it caused him great pain. He consulted with several physicians during this period, without achieving a definitive diagnosis.

**Figure 1:**
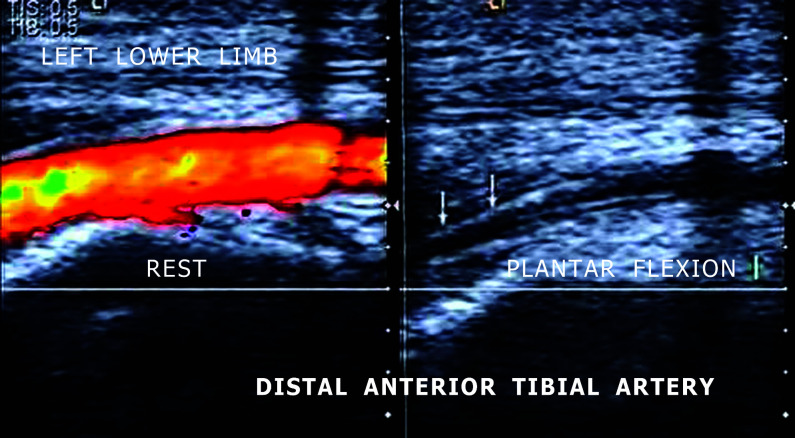
Arterial ultrasound of the distal anterior tibial artery showing artery compression during the plantar flexion maneuver.

He was instructed to consult with a vascular surgeon, who requested a vascular arterial ultrasonography (VAUS) study of the lower limbs. Bilateral arterial USV examination evaluated the common femoral, deep femoral, femoral, popliteal, anterior tibial, posterior tibial and fibular, bilaterally. At rest, the examination was normal, with no anatomic or hemodynamic abnormalities. However, since this was a case of atypical claudication, the vascular ultrasound examiner performed maneuvers to test for PAA, which were normal, with no evidence of extrinsic compression of the popliteal artery during forced dorsiflexion and plantar flexion of the foot, bilaterally. Since the complaints were more distal, primarily in the foot, the examiner repeated the same maneuvers, but this time monitoring the distal anterior tibial artery. These tests revealed narrowing of the lumen of the distal anterior tibialis artery close to the ankle, causing focal increases in flow velocity and turbulence and occasionally total abolition of flow, during the forced plantar flexion maneuver bilaterally, raising a suspicion that the cause was extrinsic compression by the retinaculum of the ankle extensor muscles (Figure 1). To better elucidate the etiology, angiotomography with contrast of the lower limbs was ordered, with axial plane slices scanned using a multidetector helicoidal technique during intravenous injection of non-ionic iodinated contrast. Images were acquired with the feet at rest and in plantar flexion (the provocative maneuver).

**Figure 2:**
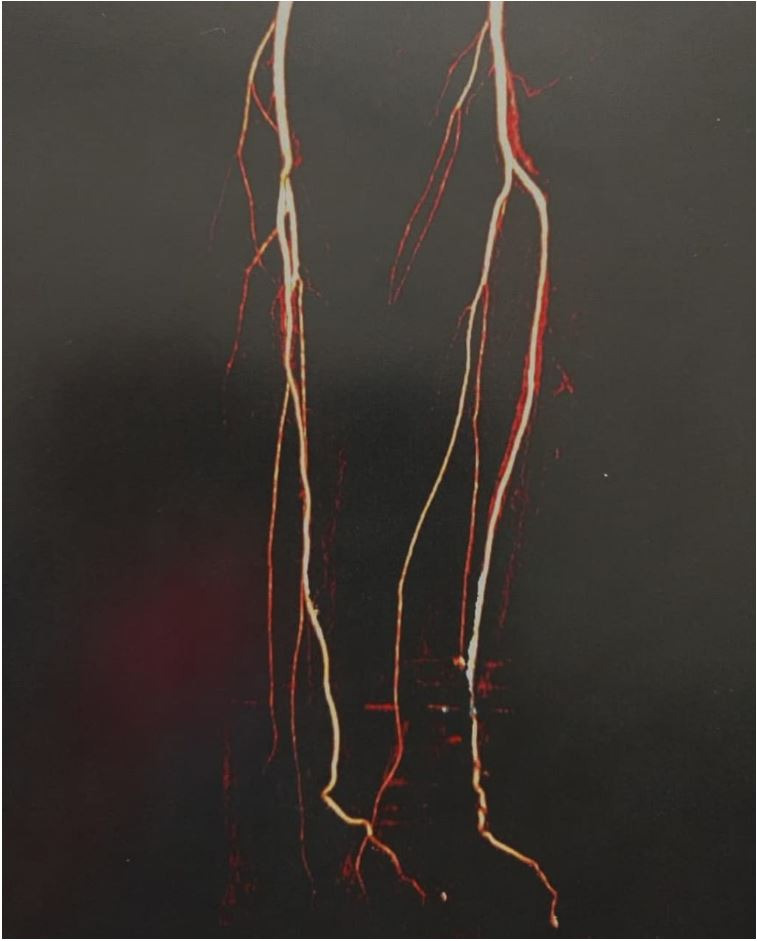
Angiotomography of the lower limbs showing the anterior tibial artery patent at rest.

**Figure 3:**
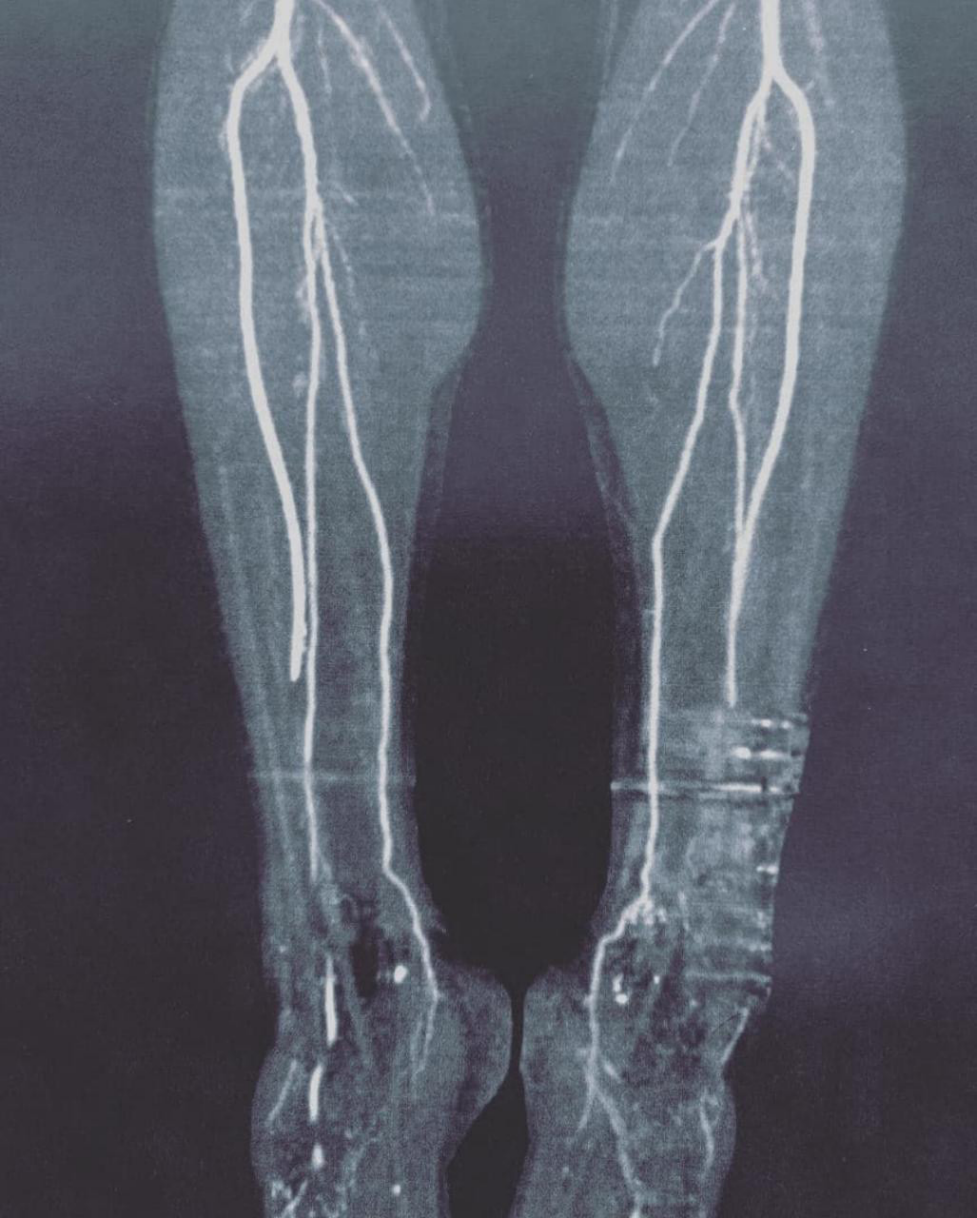
Angiotomography of the lower limbs showing compression of the tibial and dorsal arteries of the foot during the plantar flexion maneuver.

In the images acquired at rest, the anterior and dorsal tibial arteries of the feet exhibited normal opacification and caliber. However, in the images acquired with the feet in plantar flexion, the anterior and dorsal tibial arteries of the feet exhibited abnormal caliber. On the right side, the anterior tibial artery exhibited moderate stenosis of the terminal 50 mm and was collapsed in the plane of the tibiotalar joint. Two collapsed segments were identified in the dorsal artery of the right foot. On the left side, arterial collapse was identified in the distal anterior tibial artery and the entire dorsal artery of the foot (Figures 2 and 3).

Since the patient’s complaints were limiting, with a considerable impact on his quality the best approach to the case was surgical intervention with a mixed team including a vascular surgeon and an orthopedist. The patient underwent surgery on both lower limbs simultaneously, with an incision in the pre-tibial region of the distal third of the leg, dissection of the anterior tibial artery and release of the periarterial tissues, which exhibited considerable adherence and localized fibrosis. The ascending superficial fascia was released with partial release of the artery and the extensor retinaculum of the ankle was partially opened over the anterior tibial artery, liberating the arterial flow (secondary arterial angioplasty of the anterior tibialis – wide arterial pulses, with a wide pulse in the dorsalis pedis artery even with the foot in hyperextension).

The patient returned for follow-up 2 months after surgery, reporting improvement of symptoms. This case report was approved by the Research Ethics Committee at the Pontifícia Universidade Católica do Paraná (PUC-PR), under protocol number 166017.4.0000.0020.

## DISCUSSION

The striated muscles of the lower limbs require a continuous supply of oxygen to maintain metabolism. When flow is insufficient to meet metabolic requirements during exercise (or effort) because of the increased muscular demand, the anaerobic mechanism of energy release is triggered, which is responsible for the muscle pain typical of intermittent claudication (IC). Typically, IC is triggered when people with vascular disease walk specific distances. Peripheral arterial occlusive disease is the most common cause of IC, but is a condition that affects older people and is not seen in active young people free from risk factors for atherosclerosis or inflammatory disease.

When a young person with no evidence of vascular or musculotendinous disease in clinical history or physical examination complains of pain during exercise, we have a case of atypical intermittent claudication (AIC).^1^ This is characterized by feelings of discomfort triggered by certain specific movements during exercise or daily activities, with no evidence of clinical disease that could explain the complaint and which does not respond to standard treatment with rest or standard analgesics.

The characteristic clinical manifestation of popliteal artery entrapment syndrome (PAES) is pain in the foot or calf triggered by intense exercise. However, sometimes individuals may exhibit spastic claudication, paradoxically characterized by pain when walking and not during exercise of greater intensity (running).^6^ In these cases, compressive syndromes in the lower limbs should be investigated as a differential diagnosis.

Differential diagnosis for AIC with potential for surgical treatment includes: chronic compartment syndrome, medial tibial syndrome, and PAES. These conditions tend to affect high-performance athletes and football players.^1^

A study reporting on 28 years of experience at the University of Wisconsin department of surgery identified compartment syndrome as the most common cause of AC, generally bilateral, caused by muscular hypertrophy or trauma, and manifesting with symptoms of paresthesia due to nerve compression, while medial tibial syndrome and functional entrapment of the popliteal artery are less frequent. The main characteristic of chronic medial tibial syndrome is bone pain, which may be accompanied by paresthesia and cramps of the distal flexor muscles of the lower third of the leg, but not in the foot, as in our patient. In functional entrapment syndrome, the most common symptoms are cramps in the proximal part of the calf and paresthesias in the plantar region which worsen when walking or running on slopes.^1^ However, healthy individuals may also exhibit compression of the popliteal artery (functional entrapment) without exhibiting symptoms, including people who work out in gyms, performing strength exercises.^7^

A study by Almeida et al. conducted clinical and VAUS examinations of 21 athletes and 21 asymptomatic sedentary individuals, detecting 4.7% positional compression of the popliteal artery in the athletes and 9.5% in the sedentary group, showing that functional compression can occur in anyone, irrespective of physical activity levels.^8^

In the case described here, the patient complained of pains in the foot only, triggered by movements to change gear while riding a motorcycle and also while performing martial arts. It is extremely important that physicians suspect AC in young patients and remember compressive syndromes. While occlusion of the anterior tibial artery may be asymptomatic or cause few symptoms in patients with chronic arterial obstructive disease, due to the presence of collateral circulation, intermittent blockage flow in a normal artery is very symptomatic and involves a potential for endothelial injury and its consequences.

The angiosome corresponding to the anterior tibial artery is the anterior ankle and the dorsum of the foot, which are the sites that will feel pain if this artery’s flow is stopped,^9^ as in the case described here. In chronic occlusions, presence of collateral circulation and integrity of arteries of the leg compensate for the loss of blood supply and so there may not be any symptoms.

Anatomically, the anterior tibial artery arises from the popliteal artery, below the popliteal muscle, adjacent to the tendinous arches of the soleus muscle, which is a potential site of compression. It then passes through the interosseous membrane, through an oval osteofibrous space, to enter the anterior compartment. The anterior tibial artery runs along the interosseous membrane within the superior portion of the tibia, between the anterior tibialis muscles and the extensor hallucis longus.^10^ The path of the anterior tibial artery is relatively deep, following the lateral edge of the anterior tibialis muscle, along the anterior surface of the interosseous membrane, lateral to the long extensor muscle of the toe, the extensor hallucis longus.^10^ Miyamotto et al. describe a case of compression of the anterior tibial artery by the interosseous membrane, with improvement of symptoms after partial resection of the interosseous membrane, around the anterior tibial artery.^11^

Along the lower part of its path only, the anterior tibial artery adheres directly to the tibia, becoming more superficial.^10^ A consequence of this anatomy, is that ATAE may be related to tibial fractures.^12^ The anterior tibial artery continues as the dorsal artery of the foot after it passes the inferior extensor retinaculum of the ankle.^10^

Nerve compression syndrome, tarsal anterior syndrome (TAS), or anterior tarsal tunnel syndrome may all occur within the topography of the inferior extensor retinaculum of the ankle. In TAS, the symptoms are neurological, due to compression of the deep fibular nerve. Several different factors can cause TAS: traumas, fractures, subluxation, and edema.^13^ In the case described here, although the complaint described by the patient was pain in the ankles and primarily in the feet, he did not describe signs of paresthesia (deadening or tingling), but pains more similar to cramps or tiredness.

The initial imaging exam for diagnosis of compressive syndromes is VAUS,^14^ because it is a noninvasive examination that is cheap, reproducible and can be repeated during specific maneuvers when the disease is suspected. This vascular imaging exam is normal at rest, ruling out chronic obstructive arterial disease (whether atherosclerotic or inflammatory). However, complaints of pain triggered by specific movements indicate a need to extend the investigation, performing provocative maneuvers with direct real time monitoring of the artery being studied.

In the case described, compression of the anterior tibial artery was identified by VAUS in the most distal part. This is why suspicion was directed to extrinsic compression by the extensor retinaculum of the ankle as the causative factor. The diagnosis was made by VAUS with direct visualization of narrowing of the arterial lumen, causing focal increase in velocity, turbulent flow and, finally, complete collapse of the artery, causing total occlusion during the provocative plantar flexion maneuver. On relaxation, flow initially returns in vasodilation in reaction to the mechanical ischemia, followed by normalization of flow.

This hypothesis was confirmed by the angiotomography examination and, later, during surgery. During the procedure, the extensor retinaculum of the ankle was partially opened to release the compressed artery. Total resection of the retinaculum is not an option because it plays a very important anatomic role in lateral stabilization of the ankle. Although anatomy is variable, it can be observed that the retinaculum has fibers arranged in parallel to the calcaneofibular ligament and that both reach their maximum length in flexion. The retinaculum therefore prevents lateral instability in flexion.^15^

In the case described here, the patient performed martial arts with frequent movements involving kicking hard surfaces, which, when repeated, could in theory cause fibrosis of the retinaculum, reducing the space available for the anterior tibial artery. During the angiotomography imaging exam, no anatomic variations, bony calluses, or musculotendinous anomalies were identified at rest.

There are no muscle groups in the part of the ankle in which the extensor retinaculum is located that could undergo hypertrophy and cause functional entrapment of the anterior tibial artery, such as seen in functional entrapment of the popliteal artery. In functional entrapment of the popliteal artery, there is hypertrophy of the gastrocnemius, soleus, and/or plantar muscles, with no identifiable anatomic abnormality.^16^

## CONCLUSIONS

Anterior tibial artery entrapment is a unusual disease that demands active investigation for diagnosis. It is extremely important that physicians suspect this condition, enabling diagnosis and treatment. The ideal management aims to improve the patient’s quality of life.
