# Supplementary material for: Bilateral anterior tibial artery entrapment by the ankle extensor retinaculum: case report
Source: J Vasc Bras. 2020 Sep 21;19:e20200026. doi: 10.1590/1677-5449.200026 (PMC8218083; doi:10.1590/1677-5449.200026)
Supplement: Translation

# Translation: Síndrome do aprisionamento da artéria tibial anterior bilateral pelo retináculo extensor do tornozelo: relato de caso

**Authors:** Ana Luiza Dias Valiente Engelhorn, Anna Luiza Cauduro de Miranda, Luiz Eduardo Biglia, Rafaella Castilho, Sarah Folly Polonio Machado, Maurício Henrique Abrão, Carlos Alberto Engelhorn

**Affiliations:** 1 Pontifícia Universidade Católica do Paraná – PUC-PR, Curitiba, PR, Brasil.; 2 Angiolab, Curitiba, PR, Brasil.; 3 Hospital Marcelino Champagnat – HMC, Curitiba, PR, Brasil.

**Keywords:** artéria tibial anterior, claudicação intermitente, compressão arterial

## Abstract

Os aprisionamentos vasculares são raros. Nos membros inferiores, geralmente são assintomáticos, mas podem causar claudicação intermitente atípica em indivíduos jovens sem fatores de risco para aterosclerose ou doenças inflamatórias. O vaso mais frequentemente acometido é a artéria poplítea, causando a síndrome do aprisionamento da artéria poplítea (SAAP), com sintomas na região dos músculos infrapatelares. Quando o desconforto ao esforço é mais distal, deve-se pensar em outros locais de aprisionamento arterial, como a artéria tibial anterior. Neste trabalho, é relatado o caso de um paciente com claudicação intermitente nos pés devido ao aprisionamento da artéria tibial anterior (AATA) bilateral, causado pelo retináculo dos músculos extensores e diagnosticado pela ultrassonografia vascular e angiotomografia durante flexão plantar. O paciente foi tratado cirurgicamente, evoluindo com melhora dos sintomas clínicos.

## INTRODUÇÃO

A claudicação em membros inferiores é classicamente definida como uma dificuldade em caminhar e geralmente afeta indivíduos idosos com doença vascular obstrutiva crônica ou doenças ortopédicas degenerativas. Quando acomete indivíduos jovens sem aparente doença vascular ou musculotendínea identificável na história clínica e exame físico, estamos diante de uma claudicação atípica (CA)^1^.

O aprisionamento vascular nos membros inferiores pode ser causa de CA em indivíduos jovens sem fatores de risco para aterosclerose. O aprisionamento da artéria poplítea (AAP) é a condição mais prevalente, com incidência entre 0,16 e 3,5%, de etiologia congênita ou funcional, acometendo pessoas jovens, principalmente do sexo masculino, e causando sintomas envolvendo grupos musculares infrapatelares, geralmente unilateral e assintomático^2^^-^5. No entanto, quando o local do desconforto ao esforço é mais distal, localizado na região dorsal ou plantar do pé, devemos pensar em outros locais de compressão arterial mais baixos do que os tipicamente encontrados no AAP. Nessas situações, o aprisionamento da artéria tibial anterior (AATA) deve ser lembrado. O AATA é uma condição pouco diagnosticada.

O objetivo deste trabalho foi relatar um caso de claudicação atípica em um paciente jovem, com exame físico vascular arterial normal ao repouso e com diagnóstico ultrassonográfico de AATA pelo retináculo extensor do tornozelo, bilateralmente.

## RELATO DE CASO

O caso trata-se de um paciente masculino, 34 anos, não tabagista, que trabalha como motociclista fazendo entregas. Ele apresentava dor de forte intensidade localizada no tornozelo e pé esquerdo, com início há 7 anos e com característica progressiva e limitante. Na evolução, passou a apresentar dor também no pé direito, porém raramente eram simultâneas. A dor era descrita como um cansaço forte, semelhante a câimbra, desencadeada aos esforços físicos, como a prática de luta marcial e a condução da motocicleta. No entanto, podia andar longas distâncias sem sentir dor. No início, apresentava melhora com a parada da atividade e após o uso de medicação analgésica comum. Não percebeu outros sintomas associados, como mudança de temperatura e coloração dos membros.

No decorrer do tempo, o paciente teve sua qualidade de vida comprometida, afetando sua vida pessoal, profissional e social. Deixou de praticar esportes e parou de andar de motocicleta, porque causavam muita dor. Ele consultou diversos médicos nesse período até receber o diagnóstico definitivo.

Por orientação, procurou um cirurgião vascular, que solicitou uma ultrassonografia vascular (USV) arterial dos membros inferiores. O exame de USV arterial dos membros inferiores avaliou as artérias femoral comum, femoral profunda e femoral e, bilateralmente, as artérias poplítea, tibial anterior, tibial posterior e fibular. O exame ao repouso era normal, sem alterações anatômicas ou hemodinâmicas. Contudo, por se tratar de um caso de claudicação atípica, o ultrassonografista vascular realizou manobras para pesquisa do AAP, que foi normal, não havendo evidência de compressão extrínseca sobre a artéria poplítea durante as manobras de dorsiflexão e flexão plantar forçada do pé, bilateralmente. Como as queixas eram mais distais, principalmente no pé, optou-se por realizar as mesmas manobras, porém com a monitorizarão da artéria tibial anterior distal. Foi, então, identificado estreitamento do lúmen da arterial tibial anterior distal próximo ao tornozelo, causando aumento focal de velocidade e turbilhonamento de fluxo e ocasional desaparecimento total do fluxo, durante a manobra de flexão plantar forçada do pé bilateralmente, levantando a suspeita de ser causado por compressão extrínseca pelo retináculo dos músculos extensores do tornozelo (Figura 1). Para melhor elucidação da etiologia, foi solicitada uma angiotomografia com contraste dos membros inferiores cujos cortes foram obtidos no plano axial, com técnica helicoidal por multidetectores, durante a injeção endovenosa de contraste iodado não-iônico. Foram adquiridas imagens com os pés em repouso e em flexão plantar (manobra provocativa).

**Figura 1:** Ultrassonografia vascular da artéria tibial anterior distal mostrando compressão da artéria à manobra de flexão plantar.

Nas imagens obtidas em repouso, as artérias tibiais anteriores e dorsais dos pés apresentaram calibre e opacificação normais. Já nas imagens com os pés em flexão plantar, as artérias tibiais anteriores e dorsais dos pés apresentaram alteração no calibre. Do lado direito, a artéria tibial anterior apresentou estenose moderada em seus 50 mm terminais e colabamento no plano articular tíbio-talar. Na artéria dorsal do pé direito, foram identificados dois segmentos de colabamento arterial. Do lado esquerdo, foi identificado colabamento na artéria tibial anterior distal e em toda a artéria dorsal do pé (Figura 2 e Figura 3).

**Figura 2:** Angiotomografia de membros inferiores mostrando perviedade de artéria tibial anterior ao repouso.

**Figura 3:** Angiotomografia de membros inferiores mostrando compressão das artérias tibiais e dorsais do pé durante a manobra de flexão plantar.

Como as queixas eram limitantes, com grande impacto sobre a qualidade de vida do paciente, foi optado por intervenção cirúrgica com equipe mista de cirurgião vascular e ortopedista. O paciente foi submetido à cirurgia em ambos os membros inferiores simultaneamente, com incisão em terço distal da perna em região pré-tibial, dissecção da artéria tibial anterior e liberação do tecido periarterial, que apresentava muita aderência e fibrose local. Foram realizadas liberação de fáscia pré-musculatura ascendente com liberação parcial da artéria e abertura de retináculo extensor do tornozelo parcialmente sobre a artéria tibial anterior, com liberação de fluxo arterial (angioplastia arterial de tibial anterior secundária – pulso arterial amplo, com pulso em artéria pediosa amplo, mesmo na hiperextensão do pé).

O paciente retornou após 2 meses da cirurgia com melhora dos sintomas. O relato deste caso foi aprovado pelo parecer do CEP da Pontifícia Universidade Católica do Paraná (PUC-PR), sob número 166017.4.0000.0020.

## DISCUSSÃO

Os músculos estriados dos membros inferiores necessitam de aporte contínuo de oxigênio para manter o metabolismo. Quando o fluxo se torna inadequado para suprir as necessidades metabólicas durante o exercício (ou esforço) diante da demanda muscular aumentada, é utilizado o mecanismo anaeróbio para obtenção de energia, o qual é responsável pela dor muscular típica da claudicação intermitente (CI). A CI é tipicamente desencadeada durante a marcha a distâncias específicas na presença de doença vascular. A doença arterial obstrutiva periférica é a principal causa de CI, porém é uma condição que envolve pessoas idosas e não é encontrada em indivíduos jovens ativos, sem fatores de risco para aterosclerose ou doença inflamatória.

Quando indivíduos jovens sem aparente doença vascular ou musculotendínea identificável na história clínica e exame físico apresentam queixas de dor ao exercício, estamos diante de uma claudicação intermitente atípica (CIA)^1^. A CIA se caracteriza por sensação de desconforto desencadeado a certos movimentos específicos durante um exercício ou atividade cotidiana, sem evidência de doença clínica que justifique as queixas e que não responde ao tratamento padrão com repouso ou analgésicos comuns.

A manifestação clínica característica da síndrome do aprisionamento da artéria poplítea (SAAP) é dor no pé ou panturrilha desencadeada por exercícios intensos. Contudo, eventualmente os indivíduos podem apresentar claudicação espástica, caracterizada paradoxalmente pela dor ao deambular e não durante exercícios de maior intensidade (corrida)^6^.

Entre os diagnósticos diferenciais da CIA que têm potencial tratamento cirúrgico, estão: síndrome compartimental crônica, síndrome medial tibial e SAAP. São condições que geralmente acometem atletas de alta performance e jogadores de futebol^1^.

Em um estudo de 28 anos de experiência no departamento de cirurgia da Universidade de Wisconsin, é descrito que a síndrome compartimental é a causa mais comum de CA, geralmente bilateral, causada por hipertrofia muscular ou trauma, manifestando-se com sintomas parestésicos por compressão do nervo, enquanto a síndrome medial tibial e o aprisionamento funcional da artéria poplítea são os menos frequentes. O que caracteriza a síndrome medial tibial crônica é a dor óssea, podendo coexistir com parestesia e câimbra dos músculos flexores distais no terço inferior da perna e não no pé, como no nosso paciente. Já na síndrome do aprisionamento funcional, os sintomas mais comuns são câimbras na região proximal da panturrilha e parestesias na região plantar que pioram ao caminhar ou correr em declive^1^. No entanto, indivíduos normais podem apresentar compressão da artéria poplítea (aprisionamento funcional) sem a presença de sintomas, inclusive em frequentadores de academia que realizam exercícios regulares de musculação^7^.

Um estudo realizado por Almeida et al., que avaliou clinicamente e pela USV 21 atletas e 21 indivíduos sedentários assintomáticos em ambos os grupos, detectou em 4,7% dos atletas e em 9,5% do grupo dos sedentários compressão posicional da artéria poplítea, demonstrando que a compressão funcional pode ocorrer em qualquer indivíduo, independentemente da atividade física^8^.

No caso relatado, o paciente apresentava queixas de dor apenas no pé, desencadeada durante o movimento de mudar as marchas ao pilotar uma moto e ao praticar luta marcial. É de extrema importância que os médicos ao suspeitem de CA em indivíduos jovens e que lembrem das síndromes compressivas. Enquanto a oclusão da artéria tibial anterior pode passar assintomática ou pouco sintomática em pacientes com doença arterial obstrutiva crônica, devido à presença de circulação colateral, a interrupção intermitente do fluxo de uma artéria normal é muito sintomática, além do potencial de lesão endotelial e suas consequências.

O angiossoma correspondente à artéria tibial anterior é a região do tornozelo anterior e dorso do pé, local que irá apresentar dor quando há comprometimento do fluxo dessa artéria^9^, como no caso descrito. Nas oclusões crônicas, a presença de circulação colateral e a integridade das outras artérias da perna compensam o aporte sanguíneo, podendo não apresentar sintomas.

Anatomicamente, a artéria tibial anterior origina-se da artéria poplítea, abaixo do músculo poplíteo, adjacente aos arcos tendíneos do músculo sóleo, sendo um local potencial de compressão. Então, ela passa entre a membrana interóssea, por um espaço oval osteofibroso, para entrar no compartimento anterior. Na porção superior da tíbia, entre os músculos tibial anterior e extensor longo do hálux, a artéria tibial anterior percorre sobre a membrana interóssea^10^. Durante o seu curso, a artéria tibial anterior é relativamente profunda e segue a borda lateral do músculo tibial anterior, sobre a superfície anterior da membrana interóssea, lateralmente ao músculo extensor longo dos dedos e abaixo, o extensor longo do hálux^10^. Miyamotto et al. descrevem um caso de compressão da artéria tibial anterior pela membrana interóssea, com melhora dos sintomas após ressecção parcial da membrana interóssea, ao redor da artéria tibial anterior^11^.

Somente na parte inferior do curso da artéria tibial anterior, ela adere diretamente à tibial, tornando-se mais superficial^10^. Consequente a essa anatomia, o AATA pode estar relacionado às fraturas de tíbia^12^. Ao passar pelo retináculo extensor inferior do tornozelo, a artéria tibial anterior continua como artéria dorsal do pé^10^.

Na topografia do retináculo extensor inferior do tornozelo, pode ocorrer uma síndrome compressiva nervosa, a síndrome tarsal anterior (STA) ou síndrome do túnel do tarso anterior. Na STA, os sintomas são neurológicos por compressão do nervo fibular profundo. Vários fatores podem causar a STA: traumas, fraturas, subluxação e edema^13^. No caso descrito, embora a queixa de dor referida pelo paciente fosse nos tornozelos e principalmente nos pés, ele não descreveu como sensações parestésicas (amortecimento ou formigamento), mas sim como dor mais semelhante a câimbras e cansaço.

O exame de imagem inicial para o diagnóstico das síndromes compressivas é a USV^14^, pois é um exame não invasivo, barato, reprodutível e pode ser repetido e realizado durante manobras específicas quando a doença é suspeitada. O exame de imagem vascular ao repouso é normal, afastando doença arterial obstrutiva crônica (aterosclerótica ou inflamatória). No entanto, as queixas de dor desencadeada por movimento específico levam à necessidade de prosseguir a investigação, passando para a realização das manobras provocativas com monitorização direta e em tempo real da artéria que está sendo estudada.

No caso relatado, a compressão da artéria tibial anterior identificada pela USV era na porção mais distal. Por essa razão, suspeitou-se de compressão extrínseca pelo retináculo extensor do tornozelo como fator causal. O diagnóstico foi feito pela USV com a visualização direta do estreitamento do lúmen arterial, causando aumento focal de velocidade, turbilhonamento do fluxo e, finalmente, o colabamento completo da artéria, causando oclusão total durante a manobra provocativa de flexão plantar. Com o relaxamento, o fluxo retorna inicialmente em vasodilatação reativa à isquemia mecânica e há posterior normalização do fluxo.

Essa hipótese foi confirmada pelo exame de angiotomografia e posteriormente corroborada no ato cirúrgico. Durante o procedimento, foi realizada abertura parcial do retináculo extensor do tornozelo para liberação da artéria comprimida. A ressecção total do retináculo não é viável, uma vez que apresenta grande importância anatômica na estabilização lateral do tornozelo. Apesar de ter uma anatomia variável, observa-se que o retináculo apresenta fibras localizadas paralelamente ao ligamento calcâneo fibular e que ambos atingem um comprimento máximo na posição de flexão. Portanto, o retináculo tem a função de deter a instabilidade lateral na posição de flexão^15^.

No caso descrito, o paciente praticava artes marciais com frequentes movimentos que exigiam chutes em superfícies firmes, que, de forma repetida, poderiam, em tese, gerar fibrose do retináculo, diminuindo o espaço de passagem da artéria tibial anterior. Durante o exame de imagem de angiotomografia, não foram identificadas variações anatômicas, calos ósseos ou anomalias musculotendíneas ao repouso.

Na região do tornozelo em que se localiza o retináculo extensor do tornozelo, não há grupos musculares passíveis de hipertrofia que possam levar a um aprisionamento funcional da artéria tibial anterior, como aquele encontrado no aprisionamento funcional da artéria poplítea (AFAP). No AFAP, observa-se hipertrofia dos músculos gastrocnêmico, sóleo e/ou plantar, sem anormalidade anatômica identificável^16^.

## CONCLUSÃO

O AATA é uma doença pouco comum que requer busca ativa para o diagnóstico. É de extrema importância que os médicos suspeitem dessa condição, viabilizando, assim, o seu diagnóstico e tratamento. A melhor conduta tem por objetivo melhorar a qualidade de vida do paciente portador da síndrome.
